# Impact of maternal age on the risk of perineal injury and obstetric anal sphincter injury in nulliparous women

**DOI:** 10.1007/s00404-025-08286-y

**Published:** 2026-01-07

**Authors:** Emmanuel Attali, Daniel Gabbai, Ronen Gold, Asnat Groutz, Yariv Yogev, Yoav Baruch

**Affiliations:** Department of Obstetrics and Gynecology, Lis Maternity Hospital, Lis Hospital for Women’s Heath, Sourasky Medical Center, Gray Faculty of Medical and Health Sciences, Tel Aviv University, Weizman 6, Tel Aviv, Israel

**Keywords:** Maternal age, Perineal Injury, Obstetric Anal Sphincter Injury, OASI

## Abstract

**Objective:**

We aimed to evaluate the association between maternal age and the incidence of perineal injury including obstetric anal sphincter injuries (OASI) in nulliparous women.

**Study design:**

A retrospective cohort population-based study was conducted at a tertiary university-affiliated center from January 2011 to December 2020. This study included all nulliparous women with singleton pregnancies at term (37–41 weeks of gestation) in vertex and occiput anterior presentation who received epidural analgesia during labor. Exclusion criteria included stillbirth, chronic and obstetric maternal conditions (hypertension, diabetes mellitus, etc.), and deliveries complicated by instrumental vaginal delivery and episiotomy were excluded. The primary outcome was the incidence of perineal injury, defined as any spontaneous perineal tear, and/or OASI.

**Results:**

During the study period, a total of 39,596 nulliparouss women delivered vaginally at our center. Of them 3410 (8.61%) met the inclusion criteria, with 843 (24.7%) in the 20–25 age group and 2567 (75.3%) in the 30–35 age group. Significant differences were observed between the two age groups in terms of ethnicity, maternal weight before pregnancy and at delivery, gestational glucose challenge test results, gestational age at delivery, duration of the second stage of labor, and birth weight. The incidences of perineal injury and OASI were significantly higher in the 20–25 age group compared to the 30–35 age group (perineal injuries: 58% vs. 53.2%; *p* value: 0.015; OASI: 0.9% vs 0.4%, *p* value: 0.04). In the multivariable logistic regression analysis assessing the association between maternal age and perineal injury, the overall odds ratio for perineal injury in relation to the younger age category was calculated as 1.22 (CI 1.03–1.44; *p* value: 0.023), indicating a higher risk of perineal injury in the 20–25 age group compared to the 30–35 age group.

**Conclusion:**

This study suggests that younger maternal age is independently associated with an increased risk of perineal injury and OASI among nulliparous women.

## What does this study add to the clinical work

**Table:** 

In this study, younger maternal age (20–25 years) was independently associated with a higher risk of spontaneous perineal injury and obstetric anal sphincter injury compared with women aged 30–35 years among nulliparous women undergoing spontaneous vaginal delivery.	

## Introduction

Perineal lacerations and tears following delivery are extremely common, affecting up to 80% of women, with a particularly elevated occurrence among nulliparous women [1, 2]. The incidence of perineal tears differs according to the degree: first and second-degree tears are much more common than tears that involve varying degrees of anal sphincter injuries [3, 4]. Severe perineal tears can lead to short and long-term complications, such as bleeding, pain, urinary retention [5], sexual dysfunction [6], increased risk for future pelvic organ prolapse [7], and urinary or anal incontinence [8].

Only a few interventions have been proven to reduce the incidence of severe perineal tears. Applying warm compresses to the perineum during delivery, as well as perineal massage appears to reduce obstetric anal sphincter injuries (OASI) but failed to reduce second-degree tears [9]. Episiotomy, especially lateral and mediolateral, can be protective against OASI in assisted operative deliveries[10–12]; in contrast in spontaneous vaginal delivery, the risk of severe perineal trauma is lower when episiotomy is used selectively rather than routinely [13].

Identification of women who are at risk for severe perineal tears is essential to implement effective interventions for preventing such tears. Epidemiologic research regarding risk factors for perineal tears essentially focused on OASI [3, 4]. Jansson MH et al. evaluated multiple maternal and obstetric factors for perineal and vaginal tears in a cohort of 644 nulliparous women, and found no independent association between maternal age and the risk of perineal injury [14].

Despite extensive research on OASI, the specific influence of maternal age on the full spectrum of perineal trauma, remains poorly understood, especially among nulliparous women undergoing spontaneous vaginal delivery. While previous studies have addressed general risk factors for perineal injury, data specifically isolating the effect of maternal age remain limited. Therefore, in this study, we aimed to investigate the specific role of maternal age in the incidence of spontaneous perineal injury and/or OASI in nulliparous women.

### Material and methods

We conducted a retrospective large cohort study of primiparous women who underwent a spontaneous vaginal delivery over a study period of 10 years (January 2011 to December 2020). This study was conducted in a tertiary university-affiliated hospital with over 12,000 deliveries per year.

### Study population

In order to eliminate potential confounders, we included only primiparous women with a singleton pregnancy, who underwent spontaneous labor with a fetus in cephalic occipito-anterior position and epidural anesthesia and classified them by age into two groups: 20–25 years old and 30–35 years old.

A decade gap between the two age groups was chosen in order to isolate the specific impact of age on the outcome while avoiding the inclusion of women over 35, who are known to have a higher rate of peripartum maternal complications [15–17].

Epidural analgesia is used in approximately 80% of nulliparous deliveries at our institution. Limiting the sample to these cases ensured population homogeneity and minimized potential confounding, as epidural use may influence both the duration of labor and the rate of perineal tears.

Pregnancy complicated by any of the following were excluded from both groups: maternal comorbidities (for example hypertensive disorders, diabetes mellitus, maternal anemia); maternal use of systemic medication, tobacco, or alcohol; preterm birth (defined as delivery before 36 + 6 weeks); or post-term (defined as delivery after 41 + 6 weeks), as well as a non-viable fetus due to fetal demise or termination of pregnancy were excluded. Women who required oxytocin who required for labor induction, labor augmentation, or dystocia as well as those who had episiotomy or instrumental vaginal delivery, were also excluded.

### Data collection

We obtained data from our departmental electronic medical records. The following demographic and obstetrical variables were recorded: maternal age, parity, pre-gestational weight, gestational age at delivery and medical and obstetrical comorbidities. Additionally, data regarding labor and delivery, such as use of oxytocin during labor, intrapartum fever, and duration of the second and third stages as well as the need for blood products transfusion, were also obtained. In addition, data pertaining to the neonates were obtained regarding birth weight and Apgar scores. Missing data were completed specifically from personal medical charts.

### Definitions

The diagnosis and classifications of perineal tears are made by a physician in accordance with the American College of Obstetrics and Gynecology criteria [18]: first degree lacerations involve injury to the skin and subcutaneous tissue of the perineum and vaginal epithelium only; second-degree laceration extends to the fascia and musculature of perineal body; third-degree tears are defined as the involvement of the external and/or internal anal sphincter and are classified into 3A (less than 50% of external anal sphincter thickness torn); 3B (more than 50% of external anal sphincter thickness torn); and 3C (both external and internal anal sphincters torn); a fourth-degree tear involves the rectal mucosa.

Intrapartum fever was defined as at least one temperature measurement of ≥ 38.0 °C (100. 4°F) in a parturient who was afebrile at delivery admission.

Since only primigravida with epidural analgesia were included in the study, prolonged second stage was defined as a second stage of labor that lasted more than 3 h [19]. Indications for blood products transfusion included post-operative hemoglobin < 8 g/dl along with additional symptoms and signs of anemia; syncope, fatigue, pallor, reduced exercise capacity, or persistent tachycardia (> 100 bpm); or post-operative hemoglobin < 7 g/dl regardless of any additional symptoms.

The primary outcome was the incidence of perineal injury, defined as any spontaneous perineal tear, and/or obstetric anal sphincter injuries.

### Statistical analysis

Categorical variables were described as frequency and percentage. Continuous variables were evaluated for normal distribution using histogram and quantile–quantile (Q–Q) plots. Variables that were normally distributed were summarized as mean and standard deviation while skewed variables were reported as median and interquartile range. Categorical variables were compared between the two groups (women aged 20–25 years old and women aged 30–35 years old) using chi-square test or Fisher’s exact test. Continuous variables and ordinal variables were compared using independent samples T test and Mann–Whitney test.

Multivariable logistic regression was used to study the association between maternal age and perineal injury while controlling for potential confounders. Variables that were significantly different between the two groups, as well as variables that were previously published as risk factors for perineal injury or OASI (prolonged second stage, body mass index (BMI), gestational age, birth weight, Caucasian ethnicity versus other ethnicities) were analyzed. All statistical tests were two-sided and a *p* value less than 0.05 was considered as statistically significant. Software Package for Statistics and Simulation software (IBM SPSS statistics, version 27, IBM Corporation, Armonk, New York, USA 2020) was used for statistical analysis.

## Results

Overall, during the study period, 39,596 nulliparous women delivered vaginally in our department. Of them, only 3410 met our strict inclusion criteria; with 843 (24.7%) in the 20–25 age group and 2567 (75.3%) in the 30–35 age group.

Table 1 presents the maternal, obstetric, and neonatal characteristics of the study population. Notable differences were noted in baseline demographic characteristics. First, 70.5% of women in the younger age group were of Caucasian ethnicity, compared to 76.7% in the older age group (*p* value < 0.001). Additionally, the gestational age at delivery was statistically, but not clinically different between the two groups (39.4 vs 39.3; *p* value < 0.001). No differences were observed regarding pre-gestational and pre-delivery weight as well as BMI in the two groups.

**Table 1:** Demographics characteristics and pregnancy outcomes

	Age 20–25 *N* = 843	Age 30–35 *N* = 2567	*p* value
Age (years)	22.86 (± 1.66)	31.7 (± 1.57)	**0.001**
Caucasian ethnicity	594 (70.5)	1970 (76.7)	** < 0.001**
Weight before pregnancy (kg)	57 (51–63)	57 (53–63)	0.106
Weight at delivery (kg)	70 (64–77)	61 (65–78)	0.069
BMI	26.17 (24.12–28.68)	25.97 (24.09–28.28)	0.494
GCT	97 (84–112)	102 (87–117)	** < 0.001**
Gestational age at birth (weeks)	39.4 (± 1)	39.3 (± 1)	0.001
Second stage duration (min)	73 (35.5–120.5)	89 (44–139)	** < 0.001**
Prolonged second stage	51 (6.05)	229 (8.92)	**0.008**
Third-stage duration (min)	12 (10–16)	13 (10–18)	**0.001**
Neonatal weight (grams)	3228 (± 358)	3185 (± 348)	**0.002**
Male gender	402 (47.7)	1265 (49.3)	0.422
Spontaneous perineal injury	489 (58)	1365 (53.2)	**0.015**
OASI	8 (0.9)	9 (0.4)	**0.040**
Packed cells transfusion	8 (0.95)	17 (0.66)	0.397
Uterine cavity revision	11 (1.3)	63 (2.45)	**0.002**
Manual lysis of placenta	4 (0.47)	47 (1.83)	**0.002**

Data are presented as mean ± standard deviation (SD), median and interquartile range (IQR), or number (percentage), as appropriate; statistically significant values are highlighted in bold

*BMI* body mass index, *GCT* glucose challenge test, *OASI* obstetric anal sphincter injury

Regarding the neonatal characteristics, it can be noted that birthweight was significantly higher in the younger age group (3228 versus 3185; *p* value: 0.002) but no differences were seen in Apgar scores.

Labor characteristics and outcomes are also in Table 1. In the younger age group, the second stage of labor was shorter (73 min versus 89 min; *p* value < 0.001). Similarly, prolonged second stage was less frequent among younger women (6.1% vs 8.9%; *p* = 0.008). Fewer complications of the third stage of labor were observed in the younger age group, with lower rates of manual lysis and uterine cavity revision for suspected retained products of conception compared with the 30–35 years group (1.3% versus 2.45%; *p* value: 0.002 and 0.4% versus 1.83%; *p* value: 0.002, respectively).

In contrast, the incidence of perineal injury and OASI was significantly higher in this group (58% versus 53.2%, *p* value: 0.015 and 0.9% versus 0.4%; *p* value: 0.04, respectively). No difference was observed regarding the rate of packed red blood cells transfusions in the two groups.

In a multivariate logistic regression analysis model used to study the association between maternal age and perineal injury (Table 2), the overall odds ratio for perineal injury in relation to the young age category was calculated as 1.22 (CI 1.03–1.44, *p* value 0.023), reflecting a higher risk for perineal injury in the 20–25 age group compared to the 30–35 age group.

**Table 2:** Multivariable logistic regression analysis of factors associated with perineal injuries

Factor	OR	CI 95%	*p* value
Younger age group (20–25 years)	1.22	1.03–1.44	**0.025**
BMI (per 1 kg/m^2^ increase)	1.009	0.988–1.030	0.406
Gestational age (per 1 week increase)	0.985	0.914–1.060	0.683
Caucasian ethnicity	1.067	0.902–1.261	0.450
Second-stage duration (per 1 h increase)	1.001	0.999–1.003	0.154
Prolonged second stage	0.960	0.700–1.318	0.803
Neonatal weight (per 1 kg increase)	1.312	1.047–1.654	**0.018**

Statistically significant values are highlighted in bold

*OR* odds ratio

## Discussion

In the current study, we assessed the impact of maternal age on perineal injury following normal vaginal delivery. Thus, we focused on primiparous women with vertex, occiput anterior, and singleton pregnancies and compared two specific age groups that differ solely by women aged: 20–25 years old and 30–35 years old.

Our main findings revealed that the incidence of perineal injury and OASI was significantly higher in the 20–25 age group compared to the 30–35 age group. After controlling for potential risk factors, younger age was identified as an independent risk factor for perineal injury and OASI.

To our knowledge, this is one of the very few studies that have investigated the relationship between maternal age and perineal injuries of all degrees, including first- and second-degree tears as well as OASI.

We found an incidence of all-degree tears of 58% in the 20–25 age group compared to 53.2% in the 30–35 age group. This rate is higher than the rate of 40.6% reported in the studies of Samuelsson et al.[2] and Jansson et al.[14], but considerably lower than the incidence of 78.3% reported by Edqvist et al.[20]. The latter study was conducted in a Swedish setting, and differences in study populations and obstetric practices may explain the discrepancy.

In our study, we used the ACOG definitions [18] of perineal tears, while the Swedish group defined second-degree tears as vaginal tears with a depth of more than 0.5 cm. The inclusion of women with episiotomy can pose a challenge when evaluating perineal tears since it is an iatrogenic second-degree tear. Thus, in our study, we excluded women with episiotomy, in contrast to the previously cited studies.

Unlike the typical reported rate of OASI in primiparous women, ranging from 5.1 to 6.7% [1–3], the occurrence of OASI in this study is comparatively lower. However, it aligns closely with the reported rates in Israel, which range from 0.4 to 1% [21]. It is important to note that our present study excluded cases involving operative deliveries, a major risk factor for OASI. Furthermore, the incidence of OASI in our study relies on clinical diagnosis, known to be difficult, and less accurate than trans-rectal or trans-perineal ultrasound [22].

Samuelsson et al. [2] found that advanced maternal age increased slightly the risk for all perineal tears. However, in their study, they included a large and diverse population including nulliparous and multiparous women and normal and operative deliveries, and in their study, the later was not an independent risk factor for either degree of tear. Jansson et al. [14], in their prospective cohort study, did not find any significant association between second-degree perineal tears and/or OASI and maternal age, however, their study was relatively small, with 644 women in comparison to 3410 women in ours. Moreover, they compared women under 25 years old to older, and the rate of women over 35 was not reported.

Aviram et al. compared pregnancy outcomes across different maternal age groups, including women younger than 21 years, and reported a higher rate of perineal lacerations in this group compared with women aged 31–40 years [23]. However, this finding was observed only in a subgroup analysis and did not persist as an independent association after adjustment. The authors suggested that the difference might reflect higher rates of episiotomy among younger women, representing a possible selection bias related to clinical decision-making rather than a true biological effect of maternal age.

Findings regarding the association between maternal age and second-degree perineal trauma have been inconsistent. Several studies reported no significant association between maternal age and perineal injury [24], whereas others identified advanced maternal age as a risk factor for second-degree perineal tears [25–27].

Based on our study results, we hypothesize that the anatomical changes related to aging, which may be responsible for the increased rate of perinatal trauma in women of advanced maternal age, do not follow a linear pattern but rather an inverted bell curve. It is possible that during the initial aging process, decreased rigidity of perineal tissue offers some protection, whereas the subsequent loss of elasticity that occurs later in life increases the risk of perineal trauma.

Based on our study findings, we found that younger maternal age is an independent risk factor for high-grade perineal tears. This insight is important for counseling expectant mothers, as complications during pregnancy and delivery are typically associated with advanced maternal age.

More studies are needed in order to better stratify risk according to age groups and uncover the underlying mechanisms between age-related anatomical changes and perineal trauma.

### Strengths and limitations

Our study has several strengths. Our large population, even after the inclusion of only women with a singleton pregnancy, underwent spontaneous labor with fetus in a cephalic occipito-anterior position and epidural anesthesia, allowing us to focus on maternal age.

This study has several limitations. It was conducted in a single institution with a uniform protocol for the diagnosis and management of OASI, which may limit the generalizability of our findings. Restricting the cohort to women receiving epidural analgesia ensured homogeneity but may reduce applicability to other clinical settings. Moreover, due to the retrospective design, data regarding potential confounders associated with high-grade perineal tears, such as maternal position at birth and manual perineal protection, were unavailable. Finally, although a statistically significant difference in OASI rates was observed, the overall number of OASI cases was relatively small and should be taken into consideration when interpreting the results.

## Conclusion

Our study indicates that maternal age is associated with the risk of perineal injury in nulliparous women, with a higher risk observed in the younger age group. The overall odds ratio for this association is 1.22.

## Data Availability

The data are available from the corresponding author upon reasonable request.

## References

[1] Smith LA, Price N, Simonite V, Burns EE (2013) Incidence of and risk factors for perineal trauma: a prospective observational study. BMC Pregnancy Childbirth. 10.1186/1471-2393-13-59 23497085 10.1186/1471-2393-13-59 PMC3599825

[2] Samuelsson E, Ladfors L, Lindblom BG, Hagberg H (2002) A prospective observational study on tears during vaginal delivery: occurrences and risk factors. Acta Obstet Gynecol Scand 81:44–49. 10.1046/J.0001-6349.2001.10182.X 11942886 10.1046/j.0001-6349.2001.10182.x

[3] Ramm O, Woo VG, Hung YY, Chen HC, Ritterman Weintraub ML (2018) Risk factors for the development of obstetric anal sphincter injuries in modern obstetric practice. Obstet Gynecol 131:290–296. 10.1097/AOG.0000000000002444 29324610 10.1097/AOG.0000000000002444

[4] Jangö H, Langhoff-Roos J, Rosthøj S, Sakse A (2014) Modifiable risk factors of obstetric anal sphincter injury in primiparous women: a population-based cohort study. Am J Obstet Gynecol 210:59.e1-59.e6. 10.1016/J.AJOG.2013.08.043 23999415 10.1016/j.ajog.2013.08.043

[5] Mulder FEM, Rengerink KO, van der Post JAM, Hakvoort RA, Roovers JPWR (2016) Delivery-related risk factors for covert postpartum urinary retention after vaginal delivery. Int Urogynecol J 27:55–60. 10.1007/S00192-015-2768-8 26224379 10.1007/s00192-015-2768-8 PMC4715845

[6] Rådestad I, Olsson A, Nissen E, Rubertsson C (2008) Tears in the vagina, perineum, sphincter ani, and rectum and first sexual intercourse after childbirth: a nationwide follow-up. Birth 35:98–106. 10.1111/J.1523-536X.2008.00222.X 18507580 10.1111/j.1523-536X.2008.00222.x

[7] Tegerstedt G, Miedel A, Mæhle-Schmidt M, Nyrén O, Hammarström M (2006) Obstetric risk factors for symptomatic prolapse: a population-based approach. Am J Obstet Gynecol 194:75–81. 10.1016/J.AJOG.2005.06.086 16389012 10.1016/j.ajog.2005.06.086

[8] Evers EC, Blomquist JL, McDermott KC, Handa VL (2012) Obstetrical anal sphincter laceration and anal incontinence 5–10 years after childbirth. Am J Obstet Gynecol 207:425.e1-425.e6. 10.1016/J.AJOG.2012.06.055 22831810 10.1016/j.ajog.2012.06.055 PMC3484184

[9] Aasheim V, Nilsen ABV, Reinar LM, Lukasse M (2017) Perineal techniques during the second stage of labour for reducing perineal trauma. Cochrane Database Syst Rev. 10.1002/14651858.CD006672.PUB3 28608597 10.1002/14651858.CD006672.pub3 PMC6481402

[10] Lund NS, Persson LKG, Jangö H, Gommesen D, Westergaard HB (2016) Episiotomy in vacuum-assisted delivery affects the risk of obstetric anal sphincter injury: a systematic review and meta-analysis. Eur J Obstet Gynecol Reprod Biol 207:193–199. 10.1016/J.EJOGRB.2016.10.013 27865945 10.1016/j.ejogrb.2016.10.013

[11] Revicky V, Nirmal D, Mukhopadhyay S, Morris EP, Nieto JJ (2010) Could a mediolateral episiotomy prevent obstetric anal sphincter injury? Eur J Obstet Gynecol Reprod Biol 150:142–146. 10.1016/J.EJOGRB.2010.03.002 20359810 10.1016/j.ejogrb.2010.03.002

[12] De Leeuw JW, De Wit C, Kuijken JPJA, Bruinse HW (2008) Mediolateral episiotomy reduces the risk for anal sphincter injury during operative vaginal delivery. BJOG 115:104–108. 10.1111/J.1471-0528.2007.01554.X 17999693 10.1111/j.1471-0528.2007.01554.x

[13] Jiang H, Qian X, Carroli G, Garner P (2017) Selective versus routine use of episiotomy for vaginal birth. Cochrane Database Syst Rev. 10.1002/14651858.CD000081.PUB3 28176333 10.1002/14651858.CD000081.pub3 PMC5449575

[14] Jansson MH, Franzén K, Hiyoshi A, Tegerstedt G, Dahlgren H, Nilsson K (2020) Risk factors for perineal and vaginal tears in primiparous women—the prospective POPRACT-cohort study. BMC Pregnancy Childbirth. 10.1186/S12884-020-03447-0 33267813 10.1186/s12884-020-03447-0 PMC7709229

[15] Fitzpatrick KE, Tuffnell D, Kurinczuk JJ, Knight M (2017) Pregnancy at very advanced maternal age: a UK population-based cohort study. BJOG 124:1097–1106. 10.1111/1471-0528.14269 27581343 10.1111/1471-0528.14269 PMC5484369

[16] Yogev Y, Melamed N, Bardin R, Tenenbaum-Gavish K, Ben-Shitrit G, Ben-Haroush A (2010) Pregnancy outcome at extremely advanced maternal age. Am J Obstet Gynecol 203:558.e1-558.e7. 10.1016/j.ajog.2010.07.039 20965486 10.1016/j.ajog.2010.07.039

[17] Attali E, Yogev Y (2020) The impact of advanced maternal age on pregnancy outcome. Best Pract Res Clin Obstet Gynaecol. 10.1016/j.bpobgyn.2020.06.006 32773291 10.1016/j.bpobgyn.2020.06.006

[18] Practice Bulletin No (2016) 165: prevention and management of obstetric lacerations at vaginal delivery. Obstet Gynecol 128:e1-15. 10.1097/AOG.0000000000001523 27333357 10.1097/AOG.0000000000001523

[19] Gimovsky AC, Guarente J, Berghella V (2017) Prolonged second stage in nulliparous with epidurals: a systematic review. J Matern Fetal Neonatal Med 30:461–465. 10.1080/14767058.2016.1174999 27050812 10.1080/14767058.2016.1174999

[20] Edqvist M, Hildingsson I, Mollberg M, Lundgren I, Lindgren H (2017) Midwives’ management during the second stage of labor in relation to second-degree tears—an experimental study. Birth. 10.1111/birt.12267 27859542 10.1111/birt.12267 PMC5324579

[21] Baruch Y, Gold R, Eisenberg H, Amir H, Yogev Y, Groutz A (2022) Substantial obstetric anal sphincter injury during vacuum assisted delivery: an obstetrical issue or device related? J Clin Med 11(23):6990. 10.3390/jcm11236990 36498565 10.3390/jcm11236990 PMC9736983

[22] Corton MM, McIntire DD, Twickler DM, Atnip S, Schaffer JI, Leveno KJ (2013) Endoanal ultrasound for detection of sphincter defects following childbirth. Int Urogynecol J Pelvic Floor Dysfunct. 10.1007/s00192-012-1893-x 10.1007/s00192-012-1893-x 23011638

[23] Aviram A, Raban O, Melamed N, Hadar E, Wiznitzer A, Yogev Y (2013) The association between young maternal age and pregnancy outcome. J Matern Fetal Neonatal Med 26(15):1554–1558. 10.3109/14767058.2013.794212 23570233 10.3109/14767058.2013.794212

[24] Steiner N, Weintraub AY, Wiznitzer A, Sergienko R, Sheiner E (2012) Episiotomy: the final cut? Arch Gynecol Obstet 286(6):1369–1373. 10.1007/s00404-012-2460-x 22810620 10.1007/s00404-012-2460-x

[25] Woo VG, Hung YY, Ritterman-Weintraub ML, Painter CE, Ramm O (2020) A clinical risk model to predict obstetric anal sphincter injuries in laboring patients. Female Pelvic Med Reconstr Surg. 10.1097/SPV.0000000000000777 31498241 10.1097/SPV.0000000000000777

[26] Chill HH, Guedalia J, Lipschuetz M, Shimonovitz T, Unger R, Shveiky D et al (2021) Prediction model for obstetric anal sphincter injury using machine learning. Int Urogynecol J. 10.1007/s00192-021-04752-8 33710431 10.1007/s00192-021-04752-8

[27] Okeahialam NA, Sultan AH, Thakar R (2024) The prevention of perineal trauma during vaginal birth. Am J Obstet Gynecol. 10.1016/j.ajog.2022.06.021 37635056 10.1016/j.ajog.2022.06.021

